# Supplementary material for: Highly Bright Silica-Coated InP/ZnS Quantum Dot-Embedded Silica Nanoparticles as Biocompatible Nanoprobes
Source: Int J Mol Sci. 2022 Sep 19;23(18):10977. doi: 10.3390/ijms231810977 (PMC9502493; doi:10.3390/ijms231810977)
Supplement: Supplementary file 1 [file ijms-23-10977-s001.zip › ijms-1905826-supplementary.pdf]

International Journal of
*Molecular Sciences*

MDPI

*Supplementary materials*

# Highly bright silica-coated InP/ZnS quantum dot-embedded silica nanoparticles as biocompatible nanoprobes

**Kyeong-Min Ham[1,†], Minhee Kim[1,†], Sungje Bock[1], Jaehi Kim[1], Wooyeon Kim[1], Heung Su Jung[2], Jaehyun An[1,3], Hobeom Song[3], Jung-Won Kim[3], Hyung-Mo Kim[1,4], Won-Yeop Rho[5], Sang-Hun Lee[6], Seung-min Park[7], Dong-Eun Kim[1,*] and Bong-Hyun Jun[1,*]**

[1] Department of Bioscience and Biotechnology, Konkuk University, Seoul 05029, Republic of Korea
[2] Company of Global Zeus, Hwaseong, Gyeonggi-do 18363, Republic of Korea
[3] Company of BioSquare, Hwaseong, Gyeonggi-do 18449, Republic of Korea
[4] KIURI Research Center, Ajou University, Suwon, Gyeonggi-do 16499, Republic of Korea
[5] School of International Engineering and Science, Jeonbuk National University, Jeonju 54896, Korea
[6] Department of Chemical and Biological Engineering, Hanbat University, Daejeon 34158, Republic of Korea
[7] Department of Urology, Stanford University School of Medicine, Stanford, 94305, United States
* Correspondence: kimde@konkuk.ac.kr (D.-E.k.), bjun@konkuk.ac.kr (B.-H..J.)
† K.-M.H. and M.K. contributed equally to this work

Received: date; Accepted: date; Published: date

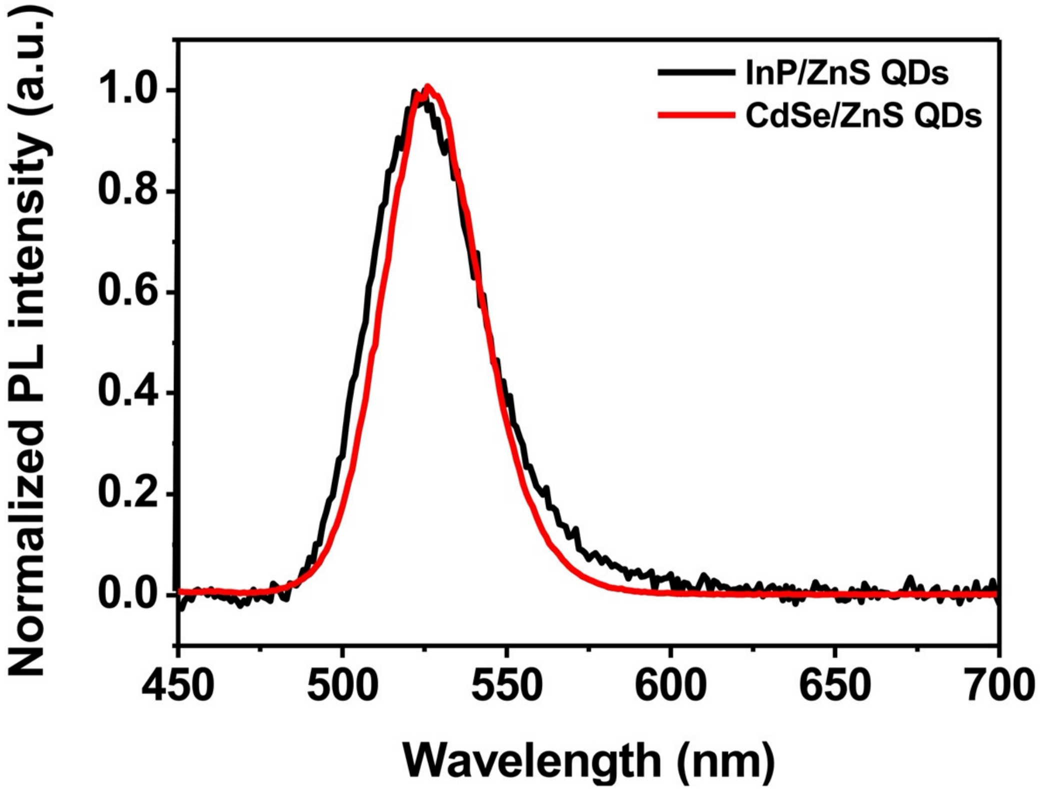


**Figure S1**. Comparison photoluminescence (PL) spectra between InP/ZnS QDs and CdSe/ZnS QDs.

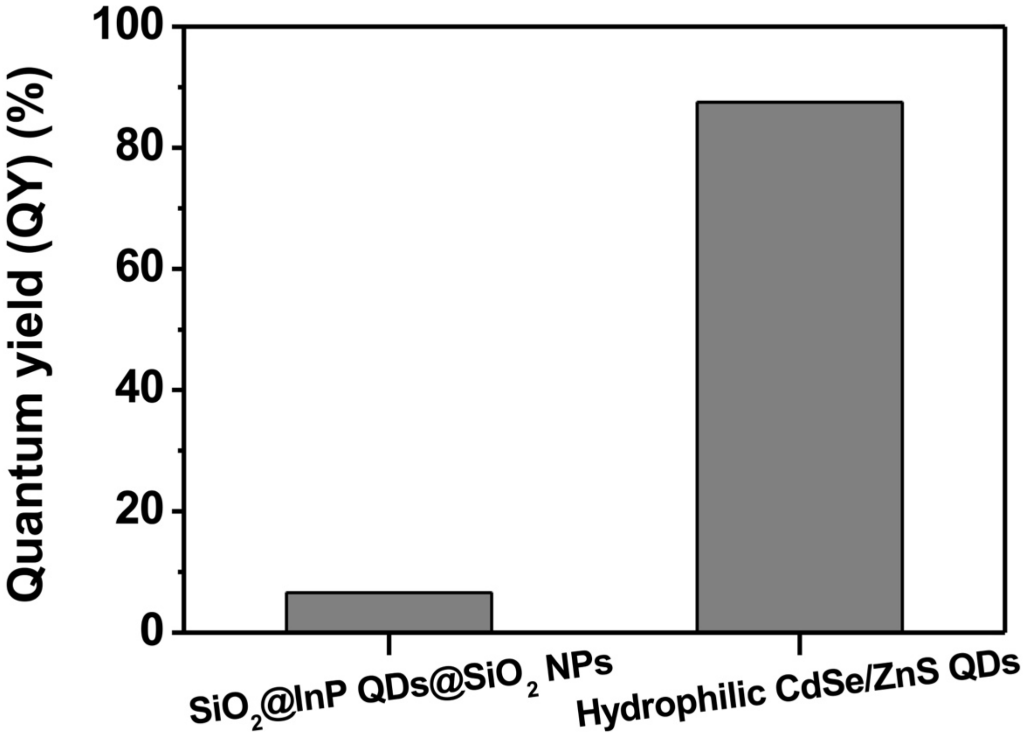


**Figure S2.** Comparison of quantum yield (QY) of $SiO_2$@InP QDs@$SiO_2$ NPs and hydrophilic CdSe/ZnS QDs. The QY of $SiO_2$@InP QDs@$SiO_2$ NPs and hydrophilic CdSe/ZnS QDs were 6.61% and 87.52%, respectively.

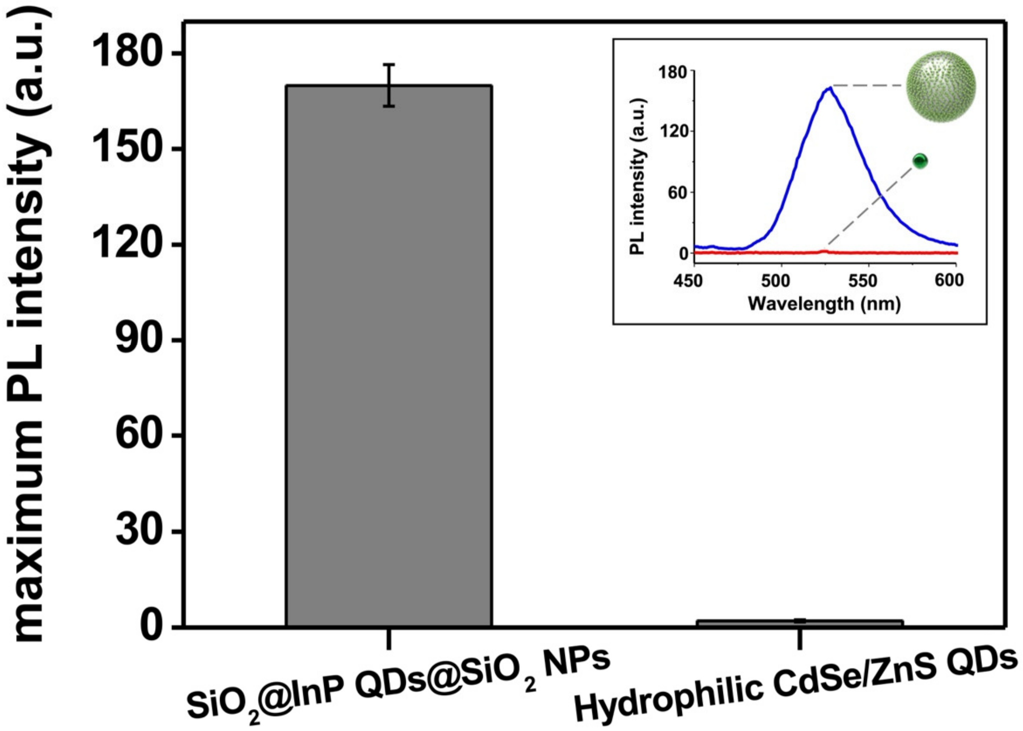


**Figure S3.** Comparison of PL intensity between $SiO_2$@InP QDs@$SiO_2$ NPs and hydrophilic CdSe/ZnS QDs. The maximum PL intensity of each particle at 527 nm emission wavelength was measured at the same concentration (2.66 × $10^{12}$ particles/mL). (**Inset**: PL intensity spectra of $SiO_2$@InP QDs@$SiO_2$ NPs and hydrophilic CdSe/ZnS QDs).

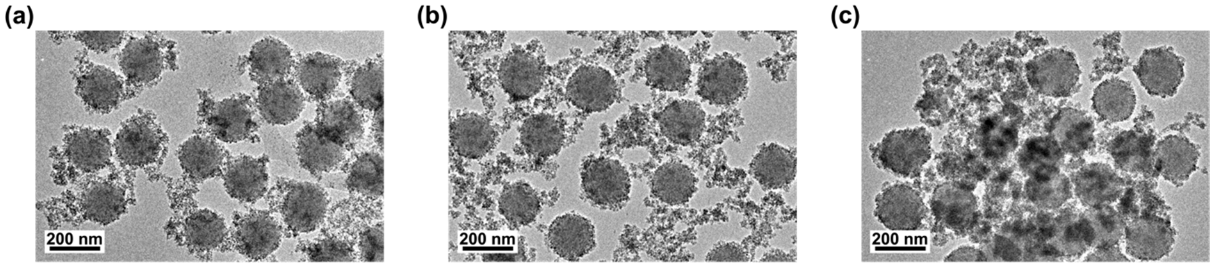


**Figure S4.** TEM images of $SiO_2$@InP QDs@$SiO_2$ NPs by amount of added QDs. The amount of added QDs were **(a)** 1.4 mg, **(b)** 2.8 mg and **(c)** 5.6 mg per 1mg of $SiO_2$ NPs.

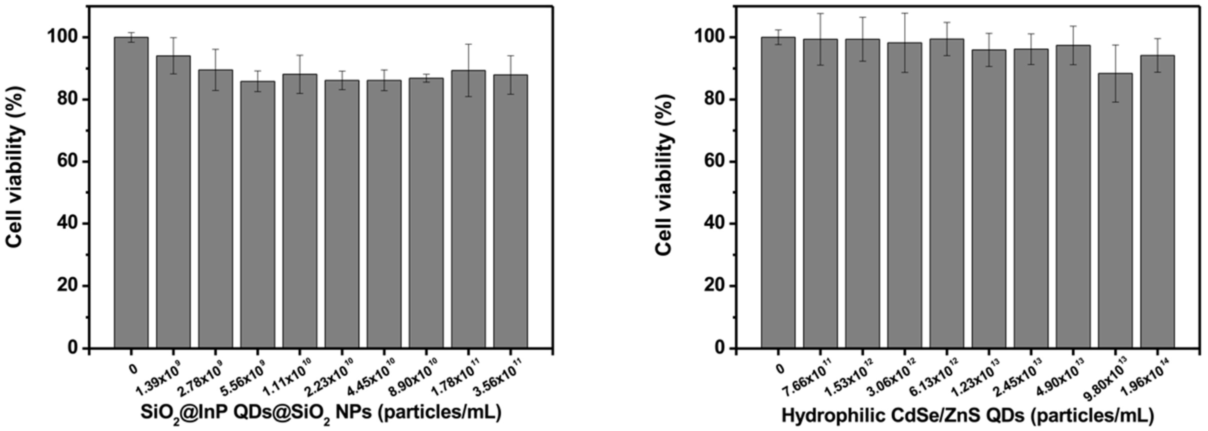


**Figure S5**. Cytotoxicity investigation of $SiO_2$@InP QDs@$SiO_2$ NPs and hydrophilic CdSe/ZnS QDs. Cell viability of A549 cells in DMEM after 24 hours incubation with $SiO_2$@InP QDs@$SiO_2$ NPs and hydrophilic CdSe/ZnS QDs.
